# Wnt/β-Catenin Expression Does Not Correlate with Serum Alkaline Phosphatase Concentration in Canine Osteosarcoma Patients

**DOI:** 10.1371/journal.pone.0026106

**Published:** 2011-10-11

**Authors:** Caroline M. Piskun, Anantharaman Muthuswamy, Michael K. Huelsmeyer, Victoria Thompson, Timothy J. Stein

**Affiliations:** 1 Department of Medical Sciences, School of Veterinary Medicine, University of Wisconsin-Madison, Madison, Wisconsin, United States of America; 2 Department of Pathobiological Sciences, School of Veterinary Medicine, University of Wisconsin-Madison, Madison, Wisconsin, United States of America; Florida International University, United States of America

## Abstract

Osteosarcoma is an aggressive malignancy of the bone and an increase in serum alkaline phosphatase concentration has clinical prognostic value in both humans and canines. Increased serum alkaline phosphatase concentration at the time of diagnosis has been associated with poorer outcomes for osteosarcoma patients. The biology underlying this negative prognostic factor is poorly understood. Given that activation of the Wnt signaling pathway has been associated with alkaline phosphatase expression in osteoblasts, we hypothesized that the Wnt/β-catenin signaling pathway would be differentially activated in osteosarcoma tissue based on serum ALP status. Archived canine osteosarcoma samples and primary canine osteosarcoma cell lines were used to evaluate the status of Wnt/β-catenin signaling pathway activity through immunohistochemical staining, western immunoblot analyses, quantitative reverse-transcription polymerase chain reaction, and a Wnt-responsive promoter activity assay. We found no significant difference in β-catenin expression or activation between OSA populations differing in serum ALP concentration. Pathway activity was mildly increased in the primary OSA cell line generated from a patient with increased serum ALP compared to the normal serum ALP OSA cell line. Further investigation into the mechanisms underlying differences in serum ALP concentration is necessary to improve our understanding of the biological implications of this negative prognostic indicator.

## Introduction

Osteosarcoma (OSA) is the most common primary bone malignancy of humans [1]. A notably aggressive disease, relapse and/or metastasis occurs in 80% of cases [2]. Five-year survival rates are approximately 80% with localized disease, but survival rate drops to roughly 30% if metastatic lesions are present [3]. Naturally occurring canine osteosarcoma is an accepted, clinically relevant animal model of human osteosarcoma; the diseases are indistinguishable grossly [3], [4] and biochemically [5]. Similar to the human disease, osteosarcoma accounts for 85–98% of all canine bone tumors [6], [7]; and pulmonary metastasis occurs in 90% of cases following removal of the primary tumor [7], [8]. As previously stated, the disease similarities between the two species have made canine osteosarcoma a valuable model for human osteosarcoma. Of additional value is the higher incidence and the accelerated biology of canine osteosarcoma compared to human osteosarcoma [5], [7]. The use of canine osteosarcoma as a comparative model allows for faster accrual of samples and performance of pre-clinical trials, with the intent to improve outcomes for both humans and canines. A better understanding of osteosarcoma is necessary, as despite advances in treatment regimens, the median survival time for either humans or dogs with osteosarcoma has not changed drastically in the last 10–20 years [1]–[3], [9]. In both species, prognosis worsens with increased serum alkaline phosphatase (ALP) concentration, correlating with shorter survival and disease-free intervals [10]–[13].

The expression of ALP is frequently used to identify cells of the osteoblastic lineage and is a hallmark of osteoblastic activity. Studies have suggested that ALP is a transcriptional target of the Wnt/β-catenin signaling pathway, as activation of this pathway in osteoblasts has been associated with increased ALP expression [14], [15]. When Wnt signaling is active, β-catenin accumulates within the cytoplasm, translocates to the cell nucleus, and associates with members of the TCF/LEF transcription factor family leading to transcription of target genes, including ALP [16].

The Wnt/β-catenin signaling pathway is crucial for normal bone development and its transient activation is required for mesenchymal stem cell (MSC) commitment to the osteoblastic lineage [17]. MSCs have been identified as a potential cell of origin in OSA development [18], and alterations in pathways involved in normal bone development may be implicated in OSA pathogenesis. However, details of the role Wnt/β-catenin signaling may play in OSA development have yet to be clarified. Support for activation of the Wnt/β-catenin pathway in OSA include the findings that nuclear and cytoplasmic β-catenin staining is present in human OSA cells [19]; that there is increased cytoplasmic and/or nuclear β-catenin expression in tissue from xenogeneic murine pulmonary metastasis [20]; and that tumorigenesis is decreased with inhibition of Wnt receptors [21], [22]. However, some report that the Wnt/β-catenin pathway is selected against in OSA development: loss of Wnt/β-catenin signaling was found to contribute towards OSA development [23]; and inhibition of Wnt/β-catenin signaling has been shown to transform human MSCs [24].

Few studies have investigated the extent of active Wnt/β-catenin signaling in canine OSA, and no studies address specifically the potential role β-catenin may have in the increased serum ALP phenotype. As a prognostic indicator, serum ALP is aligned with poor outcome, and understanding the mechanism behind this phenomenon will allow us to better understand the biological relevance of altered serum ALP concentrations in OSA pathogenesis. This insight can be used for future clinical and therapeutic interventions for both humans and animals.

Our overarching aim within this study was to determine if Wnt/β-catenin signaling is differentially activated in OSA tissue from dogs with increased serum ALP concentration. We achieved this by: (a) determining if β-catenin is differentially expressed, with respect to both quantity and cellular localization, between canine OSA patient populations with increased and normal serum ALP concentrations; and (b) determining if cell lines derived from normal and increased serum ALP OSA tissue have differential β-catenin activity as determined by: (i) the nuclear localization of β-catenin, and (ii) TCF binding site reporter plasmid activity. We hypothesized that the increased serum ALP levels associated with some canine OSA populations would be due to increased Wnt/β-catenin pathway activity.

## Results

### Evaluation of Wnt/β-catenin signaling pathway in OSA samples from patients with normal and increased serum ALP concentration using qPCR

**Figure 1A-C:**
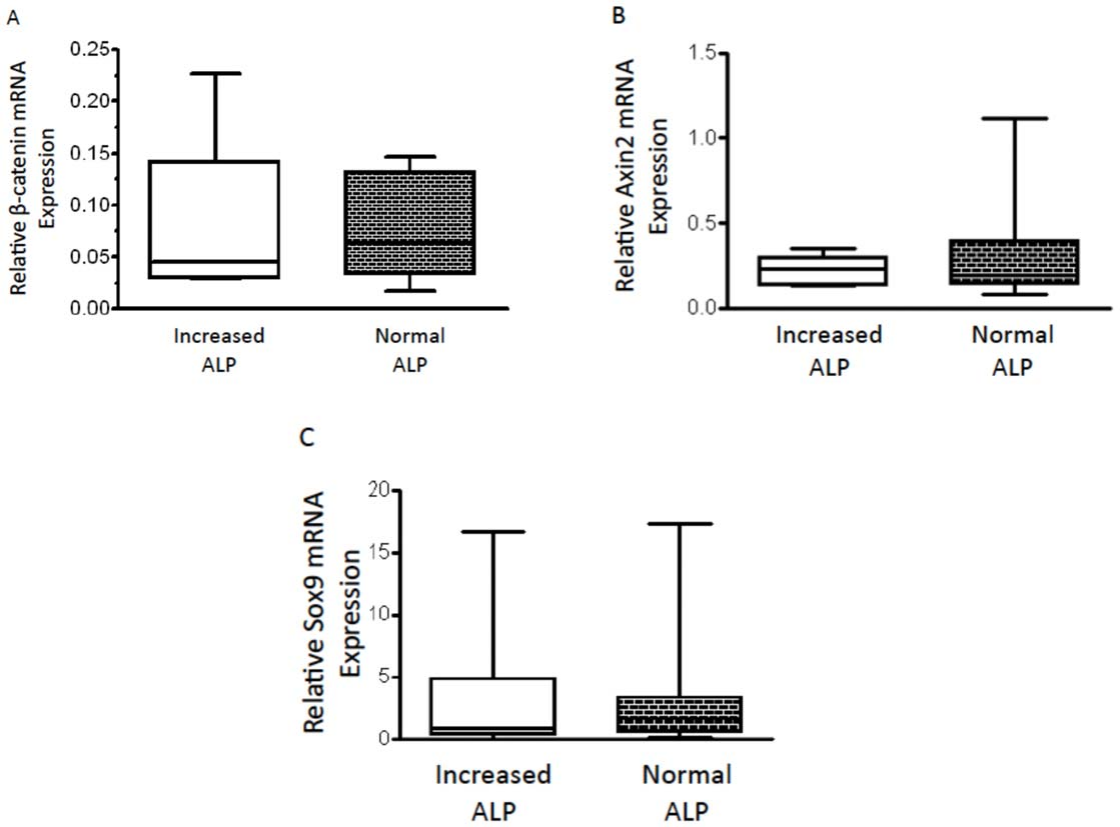
C. Evaluation of Wnt/β-catenin pathway using QPCR. (A) Relative expression of β-catenin mRNA in two canine OSA populations: increased serum ALP concentration (median  = 0.04, range  = 0.02–0.23) and normal serum ALP concentration (0.07, 0.02–0.15)(p = 0.40). (B) Relative expression of Wnt/β-catenin pathway downstream target gene Axin2 mRNA in increased serum ALP concentration (0.19, 0.08–0.35) and normal serum ALP concentration (0.20, 0.11–1.11)(p = 0.32). (C) Relative expression of Wnt/β-catenin pathway downstream target gene Sox9 mRNA in increased serum ALP concentration (0.87, 0.03–16.72) and normal serum ALP concentration (1.80, 0.20–17.24)(p = 0.24). All expression values are normalized to 18S expression and to normal, non-OSA canine bone. Comparisons made using two-tailed Mann-Whitney t-test.

Our first aim was to determine if β-catenin mRNA was differentially expressed in OSA tumor tissue from dogs with increased serum ALP concentration (n = 10) compared to OSA tumor tissue from dogs with normal serum ALP concentration (n = 9). The assignment of a patient to the increased serum ALP concentration group was based on the patient's serum ALP concentration being above the upper limit of normal as defined by the reporting institute (see Materials & Methods). The median ALP concentration for dogs with increased serum ALP that had tissue available for assessment of mRNA expression was 212 U/L (sample range: 144–733 U/L), compared to 104 U/L (sample range: 45–218 U/L) (p<0.01) for dogs with normal serum ALP. All tumor tissue mRNA was normalized to mRNA expression in normal bone tissue from a patient without OSA. There was no difference in the relative expression of β-catenin mRNA in tumor samples from dogs with increased serum ALP concentration compared to tumor samples from dogs with normal serum ALP concentration (Figure 1A).

Next, we aimed to determine if differences existed in the expression of downstream targets of the Wnt/β-catenin signaling pathway between OSA tissue from dogs with increased serum ALP concentration and those with normal serum ALP concentration. Again, qPCR was used to assess relative mRNA expression of Axin2 and Sox9, two downstream targets of Wnt/β-catenin activity. Axin2 gene expression is positively regulated by active Wnt/β-catenin signaling therefore an increase in Axin2 expression would be expected with active Wnt/β-catenin signaling. Conversely, Sox9 gene expression is negatively regulated by Wnt/β-catenin activity and a reduction in Sox9 gene expression would be expected with active Wnt/β-catenin signaling. Consistent with the lack of difference in relative expression of β-catenin mRNA between the two populations, no difference was found in the relative expression of Axin2 or Sox9 in tumor tissue associated with increased serum ALP concentration compared to normal serum ALP concentration (Figure 1B&C). Additionally, we assessed target gene expression as a continuous function of serum ALP value (non-pooled, individual analysis). For all target genes, expression did not correlate with serum ALP (β-catenin: R^2^ = 0.02, p = 0.61; Axin2: R^2^ = 0.02, p = 0.63; Sox9: R^2^ = 0.01, p = 0.70; data not shown).

### Western blot of tissue lysates from patients with normal or increased serum ALP concentration

**Figure 2:**
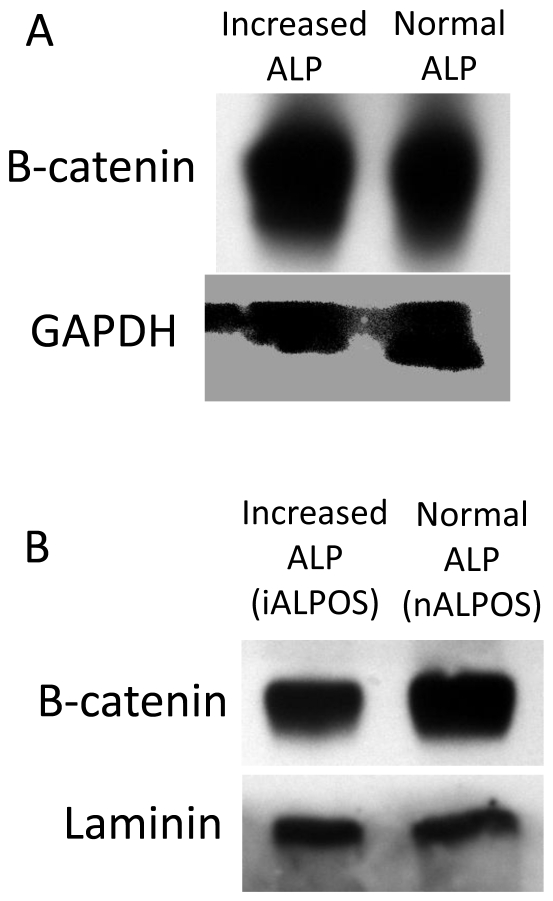
Western blot analysis of β-catenin protein expression. (A) Presence of β-catenin in canine osteosarcoma tissue lysate pooled from patients with increased (n = 5) or normal (n = 5) serum ALP concentration. Lysates were derived from a sub-population of patients used in the QPCR study. (B) Presence of β-catenin in nuclear lysate from canine osteosarcoma cell lines derived from a single patient with either increased (iALPOS) or normal (nALPOS) serum ALP concentration. GAPDH was used as a total cell lysate loading control (2A), and Laminin was used as a nuclear marker for loading control (2B).

As β-catenin is highly regulated post-translationally, protein expression of pooled tissue lysates from patients with normal (n = 5) and increased (n = 5) serum ALP concentration was assessed to confirm the mRNA expression data. Samples used for tissue lysates were a randomly chosen sub-population of those used for qPCR analysis. β-catenin was identified in the lysates from both patient populations. There was no difference in the β-catenin expression of the normal serum ALP lysate compared to the increased serum ALP lysate (Figure 2A).

### Western blot of the nuclear fraction of an increased serum ALP canine OSA cell line and a normal serum ALP canine OSA cell line derived from patient tissue

To assess the nuclear translocation of β-catenin between the two patient populations, western blot analysis was performed on the nuclear fraction of cell lysate from a single primary cell line derived from either increased serum ALP concentration patient tissue (iALPOS; serum total ALP 388 U/L, reference range: 20–157 U/L) and normal serum ALP concentration patient tissue (nALPOS; serum total ALP 124 U/L, reference range: 13–289 U/L). The nuclear fraction lysate from the iALPOS cell line displayed a slight decrease in β-catenin expression compared to the nALPOS cell line (Figure 2B).

### Luciferase Reporter Assay to determine Wnt/β-catenin pathway activity

**Figure 3:**
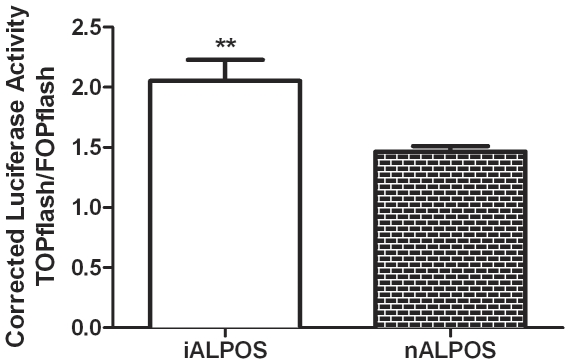
Luciferase Reporter Assay to assess Wnt/β-catenin pathway activity in osteosarcoma cell lines associated with increased (iALPOS) or normal (nALPOS) serum ALP concentration. TOPflash and FOPflash values are corrected to respective Renilla luminescence as a transfection control. iALPOS TOP/FOPflash (2.05±0.17, mean ± SEM) compared to nALPOS TOP/FOPflash (1.47±0.05) (p<0.01 using unpaired two-tailed t-test).

Both the downstream gene target qPCR and the western blot of the nuclear fraction indicate that the Wnt/β-catenin pathway is not differentially activated in patients with increased serum ALP; to further confirm this data, a TCF-responsive luciferase reporter assay was performed. β-catenin acts as a transcriptional co-factor and binds to members of the TCF/LEF transcription factor family (reviewed in 25) resulting in expression of genes downstream of the TCF sites. In the TOPflash plasmid, luciferase is downstream of several TCF sites, thus increased Wnt/β-catenin activity would be indicated by higher luciferase expression, or a higher TOP/FOP ratio. The iALPOS cell line was found to have a higher TOP/FOP ratio (2.05±0.17; mean ± SEM) compared to the nALPOS cell line (1.47±0.05; p<0.01, unpaired two-tailed t-test) (Figure 3).

### Immunohistochemistry for expression of β-catenin in tissue samples from patients with normal and increased serum ALP

**Figure 4A–C:**
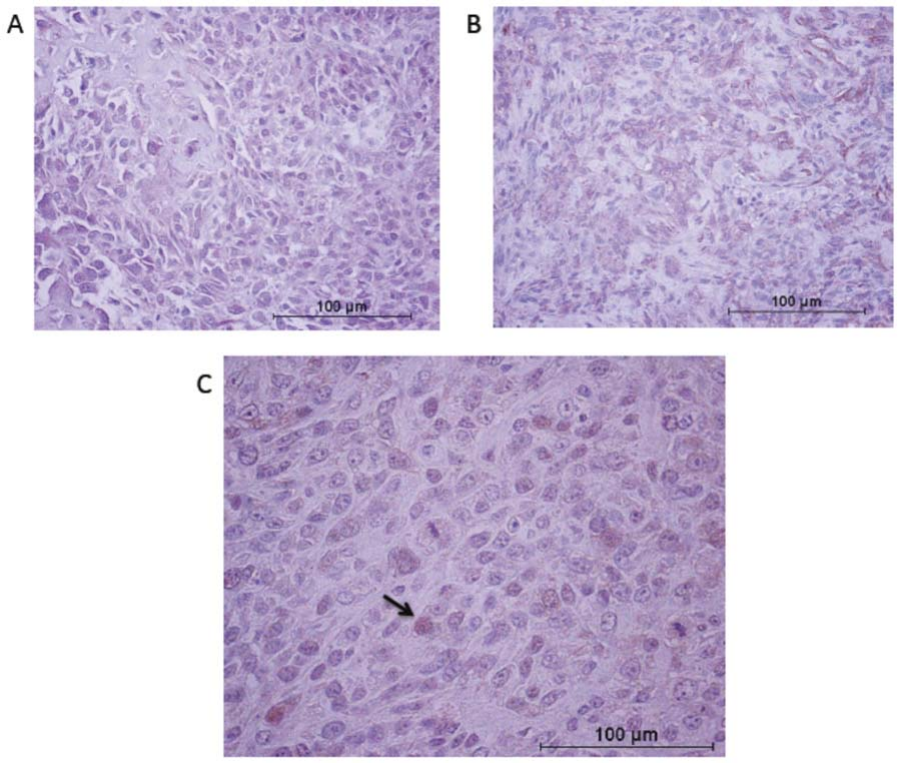
C. Immunohistochemistry to assess β-catenin protein expression and localization. (A–B) Representative images of β-catenin staining in OSA associated with increased (A) and normal (B) serum ALP concentration. (C) Photomicrograph of solitary sample with nuclear β-catenin staining; arrow indicates example of cells positive for nuclear β-catenin staining. All photomicrographs images at 400x magnification.

Given the apparent lack of differential expression or pathway activity in the small number of frozen samples and cell lines, a larger group of patients was used to confirm these findings. Immunohistochemistry was performed to detect β-catenin expression and localization in tumor tissue from patients with a known serum ALP status. As discussed above, serum ALP concentrations were determined at multiple institutions resulting in differing reference ranges. To account for differences in reference ranges, patients were assigned to the increased serum ALP concentration group based on being above the upper limit of normal for the reporting institute's reference range (see Materials & Methods). The median serum ALP concentration for dogs with normal ALP was 78 U/L (n = 32, sample range: 34–217 U/L) compared to 330 U/L (n = 24, sample range: 131->1000 U/L) for dogs with increased ALP. There was no difference in the overall β-catenin staining score between the two populations, nor was there any difference in the subcellular distribution of β-catenin staining between the two populations (Table 1, Figure 4).

**Table 1:** Characteristics of IHC staining for presence of β-catenin in canine OSA associated with increased or normal serum ALP concentration.

	Increased ALP OSA	Normal ALP OSA
Median serum ALP concentration U/L (range)^a^	330 (131->1000)	78 (34–217)
Median overall β-catenin staining score (range)^b^	3 (0–8)	6 (0–12)
Positive staining samples/total samples (%)	14/24 (58%)	22/32 (69%)
Membranous positive/total samples (%)	3/24 (13%)	9/32 (28%)
Cytoplasmic positive/total samples (%)	14/24 (58%)	21/32 (66%)
Nuclear positive/total samples (%)	1/24 (4%)	0/32 (0%)

a Difference in serum ALP concentration is significant using Mann-Whitney test (p<0.001).

b Overall β-catenin staining score is the product of percentage labelling score (0% = 0, 1-<10% = 1, 10–30% = 2, >30–60% = 3, >60% = 4) and the staining intensity score (negative  = 0, mild  = 1, moderate  = 2, intense  = 3).

## Discussion

Osteosarcoma is a notably aggressive disease, and as such, prognostic indicators are extremely useful in the clinical setting. Serum alkaline phosphatase (ALP) concentration has been correlated with overall survival times and disease-free intervals, and an increased serum ALP concentration is a negative prognostic indicator for both human and canine OSA patients [9]–[12]. However, the biological mechanism underlying the increased concentration of serum ALP in this subpopulation of OSA patients, both human and canine, has yet to be investigated. Our intent with this study was to determine whether alterations in the Wnt/β-catenin signaling pathway were part of the biological mechanism underlying this negative prognostic factor utilizing canine OSA as a comparative model for human OSA.

Cellular ALP is frequently used as a marker of osteoblasts and its expression is increased during latter stages of osteoblastic differentiation [26]. Similarly, the Wnt/β-catenin signaling pathway is upregulated during osteoblast differentiation; in fact, activation of the Wnt/β-catenin signaling pathway is essential for mesenchymal stem cells to commit to the osteoblastic lineage [17]. Without Wnt pathway activation, these cells commit to either adipogenic or chondrogenic lineages (reviewed in [16]). It is further known that the promoter region of ALP contains TCF binding sequences, which are the binding sites for β-catenin and associated transcription factors [27]. Given these facts, we hypothesized that the differences in serum ALP concentration associated with some canine OSA patients was due to differential activity in Wnt/β-catenin signaling pathway.

The major objective of this study was to determine if Wnt/β-catenin signaling is enhanced in canine OSA tissue from patients with increased serum ALP concentration compared to patients with normal serum ALP concentration. To address this, β-catenin expression was first examined at the mRNA level in frozen canine OSA samples from patients with known serum ALP concentration. No difference was found in the two patient populations at the mRNA level. This indicates that transcription of the β-catenin gene is not different between the two populations. However, β-catenin expression is highly regulated at the post-translational level and this may allow for differences to exist between the two populations at the protein level [28], [29].

Next, western immunoblots were performed on pooled tissue lysates to assess if β-catenin is differentially regulated at the post-translational level between the two populations, which would result in altered total protein expression. Total β-catenin protein expression was similar between pooled canine OSA tissue from patients with normal or increased serum ALP. This indicates that there are no differences in post-translational regulation of β-catenin between the two tumor populations that would allow for the stabilization of the protein and result in increased β-catenin expression. Constitutive expression of β-catenin is a hallmark of cancers noted for activated Wnt/β-catenin signaling, most notably colorectal carcinomas and hepatocellular carcinomas (Reviewed in [25], [30]). These cancers typically have a mutation within β-catenin itself or in one of the negative regulators of the pathway that prevents the degradation of β-catenin. Most believe the true indicator of pathway activation is denoted by the translocation of β-catenin into the nucleus (reviewed in [16]). Thus it would be possible that while the total cell quantity of β-catenin is similar between patient samples, the localization of the protein may be different, resulting in altered pathway activation.

Two methods were used to determine if the localization of β-catenin differed between increased and normal serum ALP samples: (i) western immunoblot of the nuclear fraction of cell lysate from canine cell lines derived from either an increased serum ALP patient (iALPOS) or a normal serum ALP patient (nALPOS) and (ii) IHC of biopsies taken from canine patients of known serum ALP concentration. There was a slight increase in β-catenin in the nuclear fraction of the nALPOS cell line, though no discernable differences were observed in nuclear β-catenin expression as determined by IHC for these patient populations.

The lack of difference in protein localization between the populations strongly suggests that Wnt/β-catenin pathway is not differentially activated in canine patients with increased serum ALP compared to normal serum ALP patients. However, neither western blots nor IHC are particularly quantitative, and we wanted to specifically determine the difference, if any, in pathway activity between these two patient populations. This was done using the TOPflash reporter assay, which is dependent upon the binding of transcription factors to TCF-binding sites (TOPflash) to drive luciferase expression. In this study there was a roughly 1.5-fold increase in luciferase activity in the increased serum ALP canine OSA cell line (iALPOS) compared to the normal serum ALP canine OSA cell line (nALPOS). While this value was statistically significant, the clinical significance of this difference is questionable, as there was no difference in downstream target mRNA production (neither Axin2 nor Sox9). Cell lines with mutations in the Wnt signaling pathway resulting in β-catenin stabilization demonstrate significantly increased corrected luciferase TOP/FOPflash ratios: SW480, a human colon adenocarcinoma cell line with a Wnt pathway activating mutation, has a TOP/FOPflash ratio of 10 [23]; and Mel888, a human melanoma cell line with a constitutively active β-catenin mutation, has a TOP/FOPflash ratio of 12 (data not shown). Given the basally active TOP/FOPflash ratios when compared to cell lines with known Wnt pathway activating mutations, it seems unlikely that activation of β-catenin is directly driving the differential expression of serum ALP in canine OSA.

Our study indicates that increases in serum ALP associated with some canine OSA patients is not due to differential Wnt/β-catenin signaling activity between the two populations. However, these finding are not meant to abandon the association of Wnt/β-catenin signaling with ALP expression. Rather it is possible that other intermediary signaling pathways, acting in concert with the Wnt/β-catenin pathway, are differentially activated which may amplify any Wnt/β-catenin activity. One such pathway that could be involved is the BMP/Smad signaling pathway. In this model, β-catenin prompts the expression of BMP4 and 6, which then stimulate the activation of Smad1 and Smad5 in an autocrine/paracrine fashion; this may result in differential ALP expression and/or activity [31]–[34]. Alternatively, other pathways not previously known to be associated with ALP regulation may be involved. Currently, we are performing microarray analyses to determine if other pathways are differentially activated between these two patient populations.

It is possible the differences in ALP concentration may not be due to differences at the transcriptional level. Rather, the differing ALP concentrations may be due to differences in the post-transcriptional or post-translational regulation of ALP, as it is well established that ALP expression is regulated at many levels [26].

Two other possibilities as to why no differences in β-catenin expression or activity were identified between the two canine OSA populations is perhaps the increased serum ALP concentration was not due to the bone isoform of ALP. A limitation of the current study is the inability to verify the increased serum ALP concentration was due the bone isoform of ALP. A number of analyses were performed on archived tissue, both formalin-fixed and frozen, and as such we were unable do perform iso-enzyme analysis on serum collected concurrently. However, in the selection of samples attempts were made to reduce the likelihood that increased serum ALP was due to non-bone sources by selecting only cases known to be free of corticosteroid treatment pre-operatively and concurrent liver disease. Finally, some caution must be exercised in regards to the ex vivo cell line results as only two primary canine OSA cell lines were used. However, these cell lines were used due to knowledge of the serum ALP status of the patients from which they were generated. This information, to our knowledge, is unavailable for most canine and human OSA cell lines.

To the authors' knowledge this is the first study investigating possible cellular mechanisms responsible for the differential serum ALP protein expression in canine OSA patient populations, though no significant difference in β-catenin expression or activity was found to exist between canine OSA samples associated with normal and increased serum ALP concentration. Further investigation into the mechanisms underlying differences in serum ALP concentration are important to improving our understanding of the biological implications of this negative prognostic indicator. The identification of altered signaling pathways unique to this negative prognostic factor may assist in developing therapeutic interventions based on this knowledge and potentially improve outcomes for this population of patients.

## Materials and Methods

### Ethics Statement

This study was carried out in strict accordance with the recommendations in the Guide for the Care and Use of Laboratory Animals of the National Institutes of Health. The protocol was approved by the UW-Madison's School of Veterinary Medicine Animal Care and Use Committee (Protocol: V01391-0-09-08). Owner consent was obtained prior to the collection of tumor tissue and generation of cell lines.

### Clinical Sample Selection

For experiments involving immunohistochemistry, archived tissue blocks of canine OSA associated with normal (n = 32) and increased (n = 24) serum ALP from the Pathology Service at the University of Wisconsin-Madison Veterinary Medical Teaching Hospital (UWVMTH) were identified by medical records search. Patient requirements for inclusion into this portion of the study included no treatment with corticosteroids for a period of two weeks preceding the identification of increased serum ALP concentration and liver enzyme values within normal limits with the exception of ALP. Canine OSA tissue was collected at the time of diagnostic biopsy, surgical amputation or necropsy. One investigator (AM) from the histopathology service reviewed all slides to confirm the diagnosis of OSA.

For experiments involving RNA analysis, tumor tissue was either collected from dogs at the UWVMTH or obtained from a tumor bank at Colorado State University's College of Veterinary Medicine. All tumor tissue collected at the time of a diagnostic biopsy or amputation for treatment of OSA was flash-frozen in liquid nitrogen and stored at −80°C. All tumor tissue is from dogs with known serum alkaline phosphatase concentration, drug history, and histological confirmation of OSA. Frozen normal canine bone was provided by the Comparative Orthopedics Research Laboratory at the University of Wisconsin-Madison School of Veterinary Medicine.

Cell lines were generated from the tumor tissue of dogs undergoing amputation for treatment of OSA at the UWVMTH. Patient requirements for inclusion into this study included no treatment with corticosteroids for a period of two weeks preceding the identification of increased serum alkaline phosphatase concentration and tissue collection, histological confirmation of OSA, and liver enzyme values within normal limits with the exception of alkaline phosphatase.

The determination of serum alkaline phosphatase concentration for patient samples utilized in this study occurred at multiple reference laboratories and at different times. Therefore, the normal reference ranges for serum alkaline phosphatase concentrations differed based on the reporting institute. For this reason each patient was classified as having normal or increased serum alkaline phosphatase according to the normal reference value range for that institute at the time of sampling. The reference ranges provided by institutions were as follows: 13–289, 20–157, and 20–142 U/L.

### Cell culture

Primary cell lines were generated from clinical tissue samples. Tumor tissue was surgically excised, placed on ice, and stored in phosphate-buffered saline (PBS) with PenStrepFungizone (Invitrogen, CA) to prevent microbial contamination. Mechanical and enzymatic digestion using collagenase I (200 units/ml) and DNase I (100 units/ml) was used in association with scalpel and scissors mincing, followed by filtration through a 40 mesh sieve until a single cell suspension was created. The resulting cell suspension was centrifuged for 7 min at 1400rpm. The pellet is then washed with sterile saline and re-centrifuged with the same conditions. To the pellet, 3 mL of complete modified eagle media (CMEM) is added, and the mixture is incubated in a flask with an additional 9 mL of CMEM. All cells were maintained in CMEM: MEM medium supplemented with 10% heat-inactivated cosmic calf serum, sodium pyruvate, l-glutamine, MEM vitamins, non-essential amino acids, and 1% Pen/Strep at 37°C in a humidified incubator with 5% CO_2_. All cell lines used in this experiment were beyond the 15^th^ passage.

### Quantitative PCR (qPCR)

Total RNA was isolated from clinical samples using Trizol (Invitrogen, Carlsbad, CA), and purified by PureLink RNA Mini Kit (Ambion, Life Technologies, Carlsbad, CA) according to the manufacturer's instructions. cDNA was synthesized from 250 ng of total RNA using the High Capacity cDNA Reverse Transcription Kit (Applied Biosystems, Life Technologies, Carlsbad CA) according to the manufacturers protocol. qPCR was performed using TaqMan Gene Expression Master Mix with TaqMan Gene Expression Assays (Applied Biosystems, Life Technologies, Carlsbad CA) according to the manufacters protocol on a Bio-Rad iCycler machine with a Bio-Rad iQ5 Multicolor Real-Time PCR Detection System. Assays include: canine β-catenin (Cf02667771_m1), canine Axin2 (Cf02631333_m1), and human SOX9 (Cf02631333_m1) (Applied Biosystems, Life Technologies, Carlsbad, CA). Ct values were normalized to 18S expression (4352930E). Relative difference in mRNA expression of clinical OSA samples was compared to normal canine bone obtained from the Comparative Orthopaedic Research Laboratory, School of Veterinary Medicine, University of Wisconsin – Madison, using the ΔΔCt method [35]. Gene expression of samples was measured in triplicate.

### Tissue cell lysate

Total cell lysates of ten clinical canine OSA samples (increased serum ALP n = 5 or normal serum ALP n = 5) were obtained by combining approximately 250–500 mg of tissue and 1 mL of Mammalian Protein Extraction Reagent (M-PER) in a 15 mL tissue grinder. Included samples were randomly chosen from the same patient population used for QPCR analysis. Tissue was ground until lysate appeared homogenous (5–10 min), then centrifuged for 7.5 min at 13,000rpm at 4°C. Lysates were stored at −20°C. Pooled increased serum ALP or normal serum ALP lysates were formed by combining 200 µg of protein lysate from each single sample.

### Fractionated cell lysate

Nuclear fractions of two primary cell line lysates (increased serum ALP, iALPOS, and normal serum ALP, nALPOS) were isolated using the Subcellular Protein Fractionation Kit (Pierce Biotechnology, Rockford, IL). Manufacturers protocol was used to isolate membranous and cytoplasmic fractions; the remaining pellet (nuclear fraction) was lysed with M-PER. All fractions were stored at −20°C.

### Western Blotting Analysis

Forty micrograms of protein lysate (total and nuclear) were separated by electrophoresis on a 7.5% sodium dodecyl sulphate-polyacrylamide gel at 150 V for 45 min, transferred to nitrocellulose membranes at 100 V for 1 h, then blocked with tris-buffered saline/0.05% Tween20 (TBST) containing 5% non-fat dry milk and 1% bovine serum albumin for 1 h. The membranes were probed for overnight at 4°C with either (a) mouse anti-β-catenin antibody (#610154, BD Biosciences, Bedford, MA) diluted 1∶1000 in blocking solution, (b) goat anti-GAPDH (sc-20357, Santa Cruz Biotechnology, Santa Cruz, CA) diluted 1∶200 in blocking solution, or (c) rabbit anti-laminin (ab11575, Abcam Inc, Cambridge, MA) diluted 1∶200 in blocking solution. Excess primary antibody was removed by washing three times for 5 min with TBST. Membranes were incubated with 50 ng/mL horseradish peroxidase-conjugated secondary antibody diluted in blocking solution for 1 h at room temperature, then washed three times for 5 min at TBST, and treated with SuperSignal West Pico Chemiluminescent Substrate (Thermo Scientific, Pierce Biotechnology, Rockford, IL). Blots were exposed to film, developed, and then imaged using a Gel Logic 100 Imaging System (Kodak, Rochester, NY).

### Luciferase Reporter Assay for Wnt/β-catenin activity

Cell lines were plated in a six-well plate at a density to achieve 40–50% confluency within 24 h. Cells were then transiently transfected using Lipofectamine LTX and PLUS reagents (Invitrogen, Carlsbad CA), according to the manufacturers protocol, to introduce either 2.5 µg TOPflash, or 2.5 µg FOPflash reporter plasmid, along with 0.5 µg of TK-Renilla luminescent reporter plasmid (TCF Reporter Plasmid Kit, Millipore, Temecula, CA). The TOPflash luciferase reporter plasmid contains TCF4 binding sites upstream of the luciferase gene, resulting in luciferase activity in the presence of active Wnt/β-catenin signaling, whereas the FOPflash reporter plasmid contains mutated TCF4 binding sites. The TK-Renilla plasmid serves as a control for transfection efficiency. Twenty-four hours after transfection, cells were harvested and luciferase and renilla luminescence were measured using the Dual-Luciferase Reporter Assay System (Promega, Madison, WI) on a luminometer (BioTek Synergy HT Multi-mode Microplate Reader, and Gen5 software; BioTek Instruments, Winooski, VT). The relative luciferase units for each transfection were adjusted by renilla activity in the same sample, and each corrected TOPflash luciferase value was normalized to the corresponding corrected FOPflash value. Three independent transfections were performed, with each sample assayed in triplicate.

### Immunohistochemistry

Immunohistochemical staining (IHC) was performed on slides made from archived osteosarcoma tissue to detect β-catenin protein. The process of β-catenin IHC staining and quantification of staining was performed as previously published [36]. Briefly, one investigator (AM), who was blinded to whether IHC-stained slides were from patients with normal or increased serum ALP, quantified the percentage of positively-staining tumor cells and characterized the intensity of positively-stained slides as mild, moderate or strong. Numerical values were applied to the percentage of positively-labeled cells and staining intensity with the overall staining score being the product of these two scores. Slides were imaged using a Nikon Eclipse TE2000-U microscope (Nikon Eclipse TE2000-U microscope with a Nikon Digital Camera DXM1200F using NIS-Elements F2.30 software).

### Statistical Analysis

All statistical comparisons were performed using a Mann Whitney Test with commercially-available software (Prism5, GraphPad Software, La Jolla, CA); p<0.05 was considered significant. All data are given as the median and range of values unless otherwise indicated. To determine the correlation between QPCR target gene expression and serum ALP concentration a linear regression analysis was performed using the same commercially-available software. R^2^ values and p values are reported.
